# Time discrimination and change detection could share a common brain network: findings of a task-based fMRI study

**DOI:** 10.3389/fpsyg.2023.1110972

**Published:** 2023-06-22

**Authors:** Javier Goena, Irene Alústiza, Cristina Vidal-Adroher, María Sol Garcés, Miguel Fernández, Patricio Molero, Reyes García-Eulate, María Fernández-Seara, Felipe Ortuño

**Affiliations:** ^1^Department of Psychiatry and Clinical Psychology, Clínica Universidad de Navarra, Pamplona, Spain; ^2^Instituto de Investigación Sanitaria de Navarra (IdiSNA), Pamplona, Spain; ^3^Department of Psychiatry, Basurto University Hospital, Bilbao, Spain; ^4^Colegio de Ciencias Sociales y Humanidades, Universidad San Francisco de Quito, Quito, Ecuador; ^5^Instituto de Neurociencias, Universidad San Francisco de Quito, Quito, Ecuador; ^6^Department of Radiology, Clínica Universidad de Navarra, Pamplona, Spain

**Keywords:** time discrimination, time perception, change detection, oddball paradigm, salience, cognition, cognitive control, fMRI

## Abstract

**Introduction:**

Over the past few years, several studies have described the brain activation pattern related to both time discrimination (TD) and change detection processes. We hypothesize that both processes share a common brain network which may play a significant role in more complex cognitive processes. The main goal of this proof-of-concept study is to describe the pattern of brain activity involved in TD and oddball detection (OD) paradigms, and in processes requiring higher cognitive effort.

**Methods:**

We designed an experimental task, including an auditory test tool to assess TD and OD paradigms, which was conducted under functional magnetic resonance imaging (fMRI) in 14 healthy participants. We added a cognitive control component into both paradigms in our test tool. We used the general linear model (GLM) to analyze the individual fMRI data images and the random effects model for group inference.

**Results:**

We defined the areas of brain activation related to TD and OD paradigms. We performed a conjunction analysis of contrast TD (task > control) and OD (task > control) patterns, finding both similarities and significant differences between them.

**Discussion:**

We conclude that change detection and other cognitive processes requiring an increase in cognitive effort require participation of overlapping functional and neuroanatomical components, suggesting the presence of a common time and change detection network. This is of particular relevance for future research on normal cognitive functioning in the healthy population, as well as for the study of cognitive impairment and clinical manifestations associated with various neuropsychiatric conditions such as schizophrenia.

## 1. Introduction

Perception and processing of time plays a central role in human cognition and behavior (Allman and Meck, 2012). The ability to process time is described as an implicit aspect of our relationship with the world, how we perceive it and how we communicate and relate to it (Kononowicz et al., 2018; Nakajima et al., 2018; Sato, 2022), and occupies the highest level of the hierarchy of dimensions that forms our perception of the world (Navon, 1978). Time perception is involved in relatively basic activities (such as planning-sequencing actions), in processes of higher order (such as driving a car, play a musical instrument or performing physical activity), as well as in other sensory-perceptual processes (Gheorghiu and Erkelens, 2005; Pinheiro et al., 2019; Tonoyan et al., 2022).

Despite its relevance, the study of the subjective sense of time and the brain processes linked to it has been a challenge in recent years (Drayton and Furman, 2018). The experience of time (or time perception) is an abstract concept and difficult to define. It is also difficult to point out a specific anatomical area in charge of this process (Buzsáki and Llinás, 2017). Regardless of whether it is a real concept or a construct of our internal world, the processing of time relies on a network of specialized brain areas in charge of different aspects of timing (Nani et al., 2019; Lad et al., 2020).

According to the Scalar Expectancy Theory (SET), time perception involves three different cognitive processes: an internal clock, short and long term memory, and decisional processes (Gibbon et al., 1984). Neuroimaging studies have focused on microanalysis of specific and independent brain networks related to each of the three SET subcomponents (Pouthas and Perbal, 2004; Perbal et al., 2005; Allman and Meck, 2012). Multiple meta-analyses and task-based neuroimaging studies have highlighted the presence of a broad brain network involving both cortical (supplementary motor area, premotor cortex, inferior frontal gyrus, insula, inferior parietal cortex, superior temporal gyrus) and subcortical (left putamen, right thalamus and cerebellum) areas (Wiener et al., 2010; Ortuño et al., 2011; Lad et al., 2020). The findings show dimension-related differences, depending on the modality employed by the studies (motor or perceptual) or the duration of the time discrimination (sub-second or supra-second) (Nani et al., 2019). Some areas such as the supplementary motor area are consistently recruited regardless of modality (Coull et al., 2016) while others, such as the cerebellum, are activated in sub-second timing conditions (Bareš et al., 2019). Activation/deactivation of brain circuits underlying time processing have been reported to occur during several primary cognitive functions, such as working memory and certain executive functions (Stevens et al., 2007; Wiener et al., 2010; Radua et al., 2014; Wittmann, 2015; Davalos et al., 2018; Polti et al., 2018).

Time discrimination (TD) process allows us to perceive the duration of an event, that is, to calculate how much time has elapsed during a time interval. Thus, TD is basically and essentially a cognitive process related to the detection of change. Any non-temporary task requires processing changes, such as variations in the presentation of perceived stimuli or fluctuations in the level of demand. It is therefore expected to involve temporality processing and the involvement of the brain networks of temporality that support it (Livesey et al., 2007; Ortuño and Alústiza, 2014).

There is a growing consensus in the field of neurosciences about the fact that behavioral and cognitive functions are emergent properties, meaning that the combination and coordination of different brain regions give rise to new and complex functions that cannot be understood by simply looking at the individual regions roles (de Schotten and Forkel, 2022). These emergent properties are the result of the interactions and integration of different brain areas working together, which enables the performance of other functional roles such as perception, memory, decision-making, and more (Pessoa, 2022). Regarding time perception, several studies have documented this hypothesis, showing that the network associated with TD is involved in other processes, such as change detection (Stevens et al., 2000, 2007; Menon and Uddin, 2010; Alústiza et al., 2018; Justen and Herbert, 2018; Garcés et al., 2021) or cognitive control (Radua et al., 2014; Alústiza et al., 2017; Garcés et al., 2021), especially when execution of a task requires increases in cognitive effort (Alústiza et al., 2016; McTeague et al., 2016, 2017). Cognitive effort is an aspect of every cognitive process; it reflects the level of difficulty of a cognitive task and the resulting mental effort that a person must apply to achieve the cognitive goal (Radua et al., 2014).

The salience network, in charge of detecting salient or novel stimulus and guiding flexible behavior by regulating relevant functional networks, involves anterior insula, anterior cingulate cortex, amygdala, superior temporal gyrus, parietal cortex, ventral striatum and ventral tegmental area (Menon, 2015). One of the traditional paradigms to study this network involved in change detection is the oddball detection (OD) paradigm. This consists of an experimental technique in which a series of standard repetitive stimuli (auditory or visual) are presented and occasionally an infrequent stimulus (known as *oddball* or *deviant*) is included which evokes a reaction in the subject (Squires et al., 1975; Polich, 1986).

For several years, there has been growing research interest in temporal perception and its involvement in other cognitive processes (Head et al., 2008), both in healthy subjects and in relationship with psychopathology (Buhusi and Meck, 2005; Davalos et al., 2018). Both time and change processes are cognitive functions acquired gradually throughout human neurodevelopment (Buhusi and Meck, 2005; Brannon et al., 2008; Merchant et al., 2013). The interrelationship between TD and change detection processes may explain various aspects of normal and pathological cognition. With respect to normal cognition, we hypothesize the existence of a common *time and change detection* brain network that supports multiple non-temporal tasks. In a previous study, we conducted two independent signed differential mapping (SDM) meta-analysis of fMRI studies using timing and OD paradigms and a multimodal meta-analysis to elucidate whether there are brain regions common to these two processes, finding that the pattern of brain activation partially coincided (Garcés et al., 2021). These findings support the hypothesis that change detection paradigms, such as OD, could be related to a basic underlaying TD function and, eventually, to other non-temporary task and cognitive control processes, as far as they both implicate change.

Regarding pathological conditions and considering TD as a primary cognitive domain, this *time and change detection* network could clarify and explain some common features of brain disorders of different natures and pathophysiology. Multiple neuroimage studies and reviews point to the involvement of brain regions related to temporality and cognitive control functions in schizophrenia (Radua et al., 2014; de Montalembert et al., 2015; Alústiza et al., 2016, 2017; Ciullo et al., 2016; Lošák et al., 2016), neurodegenerative processes (Alústiza et al., 2016; Lad et al., 2020; Cope et al., 2022) and other neuropsychiatric conditions (Piras et al., 2014; Wise and Barnett-Cowan, 2018).

In this study we focus on brain activity involved in TD processes in neurotypical (healthy) people. Could TD and OD paradigm based tasks reveal the existence of a common brain network? Is this common network involved in processes of higher cognitive effort? To try to answer these questions, we designed our own experimental auditory test to assess TD and OD paradigms. In this proof-of-concept study our main goal is to describe the pattern of brain activity involved in TD and OD paradigms and in processes requiring higher cognitive effort. We hypothesize that the activation pattern of the TD network coincides to some degree with that of brain activity during the performance of OD and with the activation patterns of other brain networks involved in cognitive control processes.

## 2. Materials and methods

### 2.1. Design

We designed an experimental, descriptive, cross-sectional and quantitative study. Participants carried out tasks whilst undergoing fMRI of the brain. Tasks were based on different established paradigms (with different conditions) but were adapted so that subjects could carry them out within the fMRI machine. The objective was to scan brain activity generated during execution of experimental tasks.

### 2.2. Subjects

Recruitment was of healthy individuals. Inclusion and exclusion criteria are listed in Table 1. Possible participants completed an initial interview, which included sociodemographic information, and underwent detailed clinical assessment. All participants gave informed written consent to the participation. The study protocol was approved by the Research Ethics Committee of the University of Navarra.

**Table 1:** Exclusion and inclusion criteria.

Inclusion criteria
Age between 18 and 65 years old
Spoken language: Spanish

Exclusion criteria
Intelligence quotient (IQ) less than 80; Hearing loss; Psychiatric disorder; Relevant neurological or medical illness; History of traumatic brain injury with loss of consciousness for more than one hour; Contraindications for MRI (metal prostheses, pacemakers, claustrophobia, etc.)

The study sample was formed by 14 healthy volunteers (6 women and 8 men; mean age was 36 years, with standard deviation of 9.29 years), right-handed (except one left-handed subject). None of the volunteers had personal or first-degree family history of psychosis, or any other mental disorder (common or severe).

From the original sample (*n* = 14), a total of 13 participants successfully completed the study. The data from one of the subjects could not be analyzed due to technical problems, and therefore this subject was excluded from the final results of the study.

### 2.3. Experimental task

We designed our own test to assess TD and OD paradigms. In both paradigms, we built a cognitive control component by introducing two levels of difficulty (*difficult* and *easy*). Thus, participants were required to do two tasks (TD and OD) at two levels of difficulty: a total of four tasks (*TD easy*; *TD difficult*; *OD easy*; *OD difficult*).

#### 2.3.1. Time discrimination (TD) paradigm

**Figure 1:**
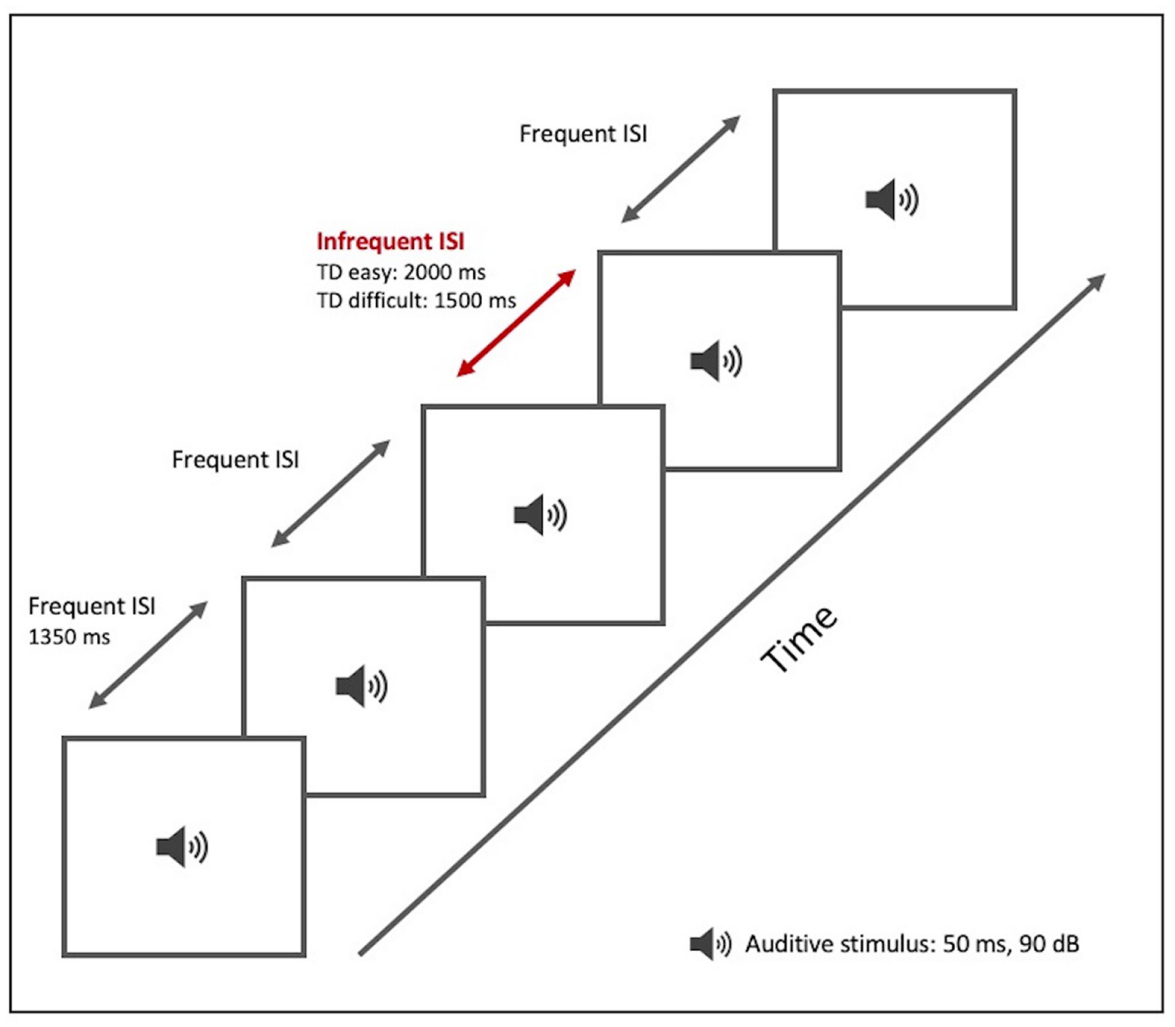
TD paradigm test tool. Graphical representation.

We designed a TD test (Table 2; Figure 1) based on the repetition of a sound (a pure sine wave at a frequency of 1,000 Hz and a sound pressure level of 90 dB/SPL) of 50 ms duration. The subject was required to indicate a change in the length of the silence between the sound beeps: the inter-stimulus interval (ISI). The test included both frequent and infrequent ISIs. The frequent ISI was 1,350 ms long, while infrequent ISIs varied in duration. The test was divided into two levels of difficulty:

- At the easy level (*TD easy*), we included a total of 21 auditory stimuli and 20 ISIs, with 80% being the frequent ISI and 20% (positions 3, 7, 13, and 17) being infrequent (2000 ms long).
- At the difficult level (*TD difficult*), we included 23 sounds and 22 ISIs. The frequent ISI had a frequency of occurrence of 73.3%, while 22.7% (positions 4, 7, 13, 16, 21) of the ISIs were infrequent, with a duration of 1,500 ms.

**Table 2:** TD test tool.

TD paradigm	Duration	
Auditory stimulus	50 ms/1,000 Hz	
Frequent ISI	1,350 ms	
Infrequent ISI	2,000 ms (Easy)	1,500 ms (Difficult)

#### 2.3.2. Oddball detection (OD) paradigm

**Figure 2:**
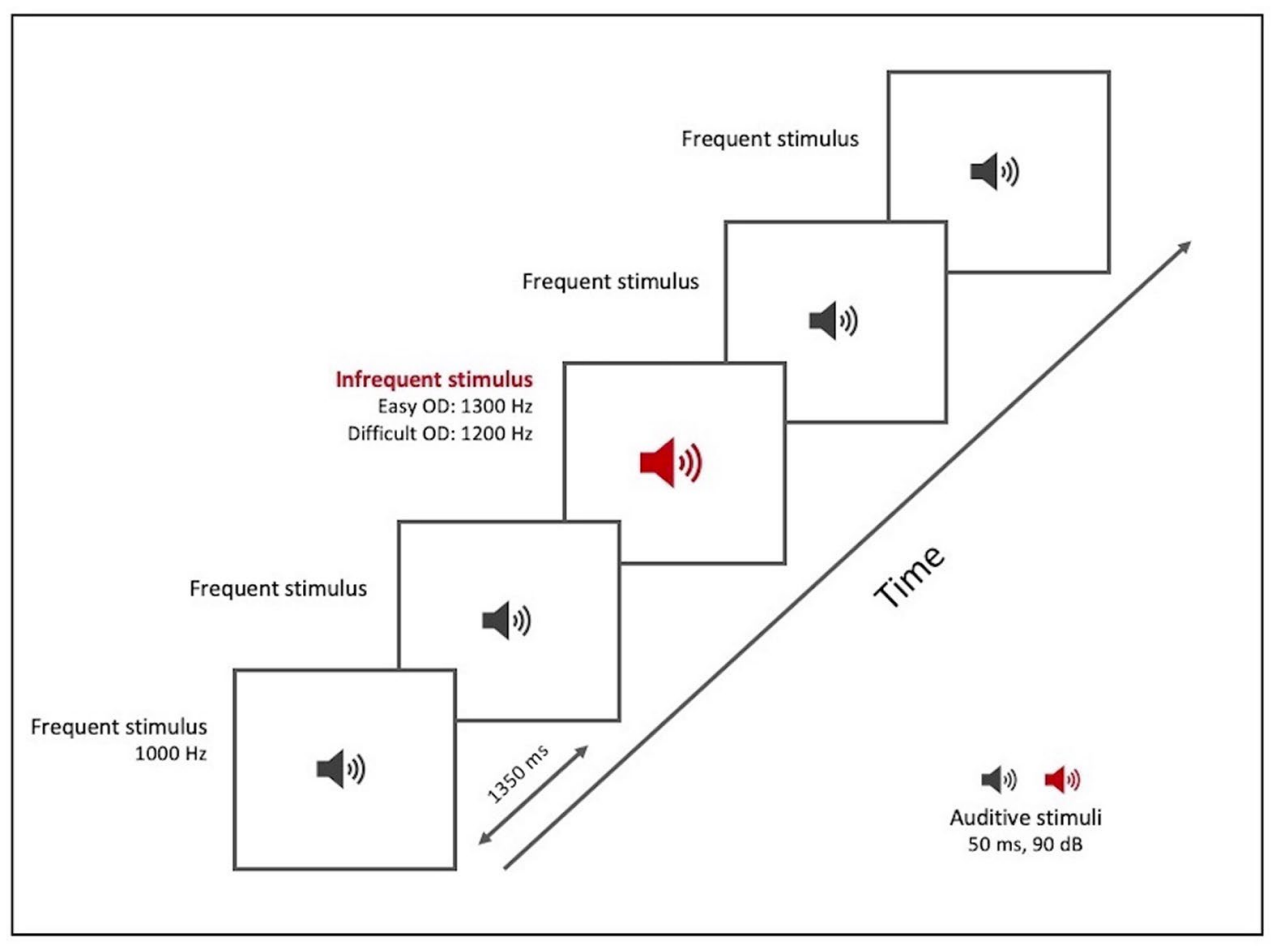
OD paradigm test tool. Graphical representation.

The OD test (Table 3; Figure 2), like the TD test, was based on repetition of a sound (a pure sine wave at a sound pressure level of 90 dB/SPL) of 50 ms duration, with an ISI of 1,350 ms. The subject was required to detect and indicate change in pitch of the sound. The test included a total of 22 stimuli, both frequent and infrequent. The frequent stimulus (with an 80% probability of occurrence) had a frequency of 1,000 Hz. The infrequent stimulus (with a 20% probability) was divided into two levels of difficulty:

- At the easy level (OD easy), the infrequent stimulus had a frequency of 1,300 Hz and appeared at positions 5, 8, 13, 17, and 19
- A t the difficult level (OD difficult), the infrequent stimulus had a frequency of 1,200 Hz and appeared at positions 4, 7, 12, 16, and 19.

**Table 3:** OD test tool.

OD paradigm	Frequency	
Frequent stimulus	1,000 Hz	
ISI	1,350 ms	
Infrequent stimulus	1,300 Hz (Easy)	1,200 Hz (Difficult)

#### 2.3.3. Task presentation

The above tasks were adapted and conditioned for fMRI. Tasks were done in blocks of 9 min duration. There was one block for the TD paradigm and another for the OD paradigm. After a rest period, the two blocks were repeated and so in total the test took about 40 min and we obtained 18 min of BOLD data for each paradigm (TD and OD). We alternated the order of the paradigms between participants, with some starting with the TD paradigm and others with the OD paradigm (generating two possible order of presentations: TD-OD-TD-OD or OD-TD-OD-TD).

Each block comprised 10 iterations of a single task (five easy and five difficult, alternating). Before each individual task, subjects did a “control task” in which they had to perform a non-discriminatory action. The stimulus sequence for the control task was the same as that for the subsequent discrimination/detection task. After a rest period, the two blocks were repeated, and so in total the test took about 40 min and we obtained 18 min of BOLD data for each paradigm (TD and OD).

**Figure 3:**
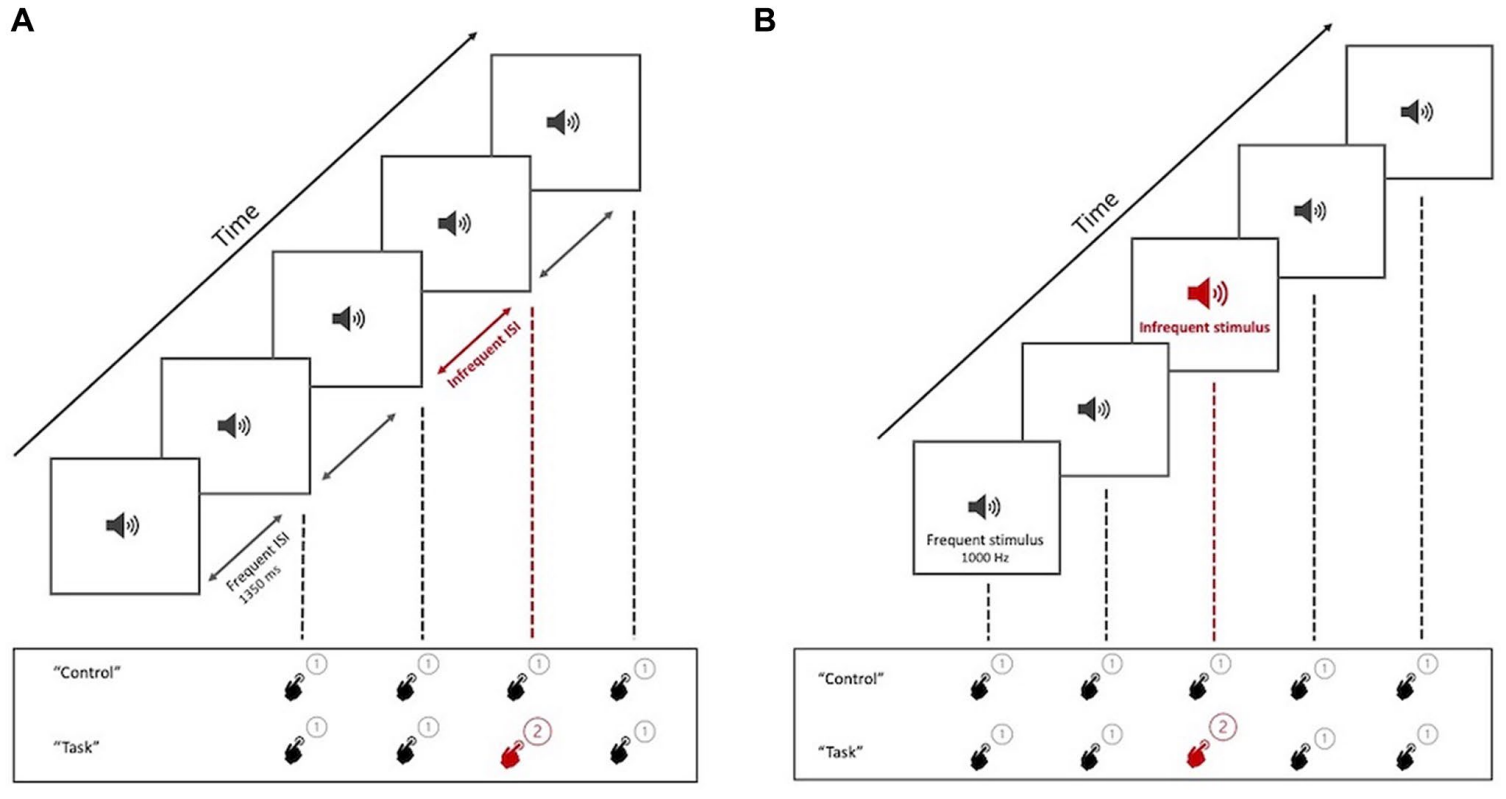
Graphical representation of the task presentation. **(A)** TD paradigm test, with the two tasks to be performed by the participant (Control and Task). **(B)** OD paradigm test, with the two tasks to be performed by the participant (Control and Task). When hearing the command “control,” participants press “1” regardless of the perceived stimulus (non-discriminatory activity). At the command “task,” participants must press “2” for the infrequent trait.

Each block of trials began with an oral statement of the name of the paradigm (“time discrimination” or “oddball detection”) and a brief reminder of the action to be performed (pressing one of two buttons on a simple hand-held controller). Upon hearing the command “control,” subjects were to press the “1” button after each beep in the stimulus sequence, regardless of its characteristics, without making any discriminative effort. On hearing “task,” they were to press “1” after hearing the frequent form of the stimulus and “2” after hearing an infrequent form of the stimulus (i.e., a longer interval than usual, in TD, or higher frequency than usual, in OD) (Figure 3).

All participants did a training session of the test on a computer, prior to the fMRI session.

### 2.4. fMRI scanning

Studies were performed on a 3 Tesla MRI scanner (Siemens Skyra; RRID:SCR_020530) using a 20-channel head coil. A T2* weighted echo-planar imaging (EPI) sequence was employed to acquire the BOLD fMRI images. Each volume consisted of 46 slices, with a slice thickness of 3 mm and slice gap of 15%. Time interval between two consecutive acquisitions (TR) was 3 s and echo time (TE) was 30 ms. In-plane image resolution was 3.4 × 3.4 mm^2^, field-of-view (FOV) was 220 × 220 mm^2^, and flip angle was 90°. An anatomical image was also acquired using a 3D T1-weighted MPRAGE sequence with imaging parameters: TR = 2,320 ms, TE = 3.68 ms, inversion time (TI) = 1,170 ms, FOV = 208 × 256× 192 mm^3^, flip angle = 8°, number of slices = 192, and voxel size = 1 mm isotropic.

### 2.5. fMRI data analysis

BOLD images were pre-processed and analyzed using “Statistical Parametric Mapping” software, version SPM12 (RRID:SCR_007037), in MATLAB (RRID:SCR_001622). All EPI volumes were realigned to the mean and co-registered to the anatomical image. The anatomical image was then normalized to the Montreal Neurological Institute (MNI) template and the normalization parameters were applied to the EPI images. In this step, the EPI images were resampled to an isotropic voxel size of 2 mm. A 3D Gaussian smoothing filter of 8 mm full-width at half maximum was applied to the EPI images.

Statistical analysis was performed at two levels. At the first level, individual subject data were entered into a general linear model (GLM) (Friston et al., 1994). The design matrix was composed of 16 task/control regressors, 6 movement parameters as nuisance regressors and a constant regressor. The 16 task/control regressors model the occurrence of an event type. There were 8 event types: 2 paradigms (TD and OD) x 2 difficulty levels (*easy* and *difficult*) x 2 conditions (*task* and *control*). The regressors were convolved with a hemodynamic response function (canonical HRF). After model estimation, we calculated statistical contrasts in order to compare brain activation patterns.

For each individual, we performed the following t-contrasts with different objectives:

- To assess the brain activity evoked by each of the paradigms:

1. *TD task contrast (TD task > TD control).*
2. *OD task contrast (OD task > OD control).*

- To determine the differential/specific activation pattern of each of the paradigms:

1. *TD specificity contrast (TD task > OD task).*
2. *OD specificity contrast (OD task > TD task).*

- To measure the cognitive effort component in each of the paradigms:

1. *TD effort contrast (TD task difficult > TD task easy).*
2. *OD effort contrast (OD task difficult > OD task easy).*

- To analyze the differential/specific activation pattern of the TD paradigm in the cognitive effort component:

1. *TD specificity in cognitive effort contrast [(TD task difficult > TD task easy) > (OD task difficult > OD task easy)].*

The first level analysis was followed by group inference using the random effects model (Penny and Holmes, 2003). Random-effects group t-maps were generated for each of the first-level contrasts by applying a one-sample t-test for the contrast parameter values of all the subjects at each voxel. The significance level was set at a cluster-corrected *p* value of 0.05, with a cluster-generating threshold of 0.001. If map-wise analysis at this corrected level of significance failed to identify areas of activation, results were evaluated using an uncorrected p value of 0.001 with a minimum cluster size of 50.

Additionally, conjunction analysis was performed to determine areas of overlap between *TD task* and *OD task* (contrasts 1 and 2). This was done in two steps: first, thresholded activation maps were saved for both contrasts (i1 for TD and i2 for OD, respectively). Subsequently the imcalc tool was used in SPM with the following equation: (i1 > 0) + 2*(i2 > 0). The result was a new image with a value of 3 in areas activated by both tasks, a value of 1 in areas activated by TD only and a value of 2 in areas activated by OD only.

## 3. Results

The analysis of the fMRI data revealed distinct patterns of brain activity in response to the different planned contrasts.

### 3.1. TD and OD activity

**Figure 4:**
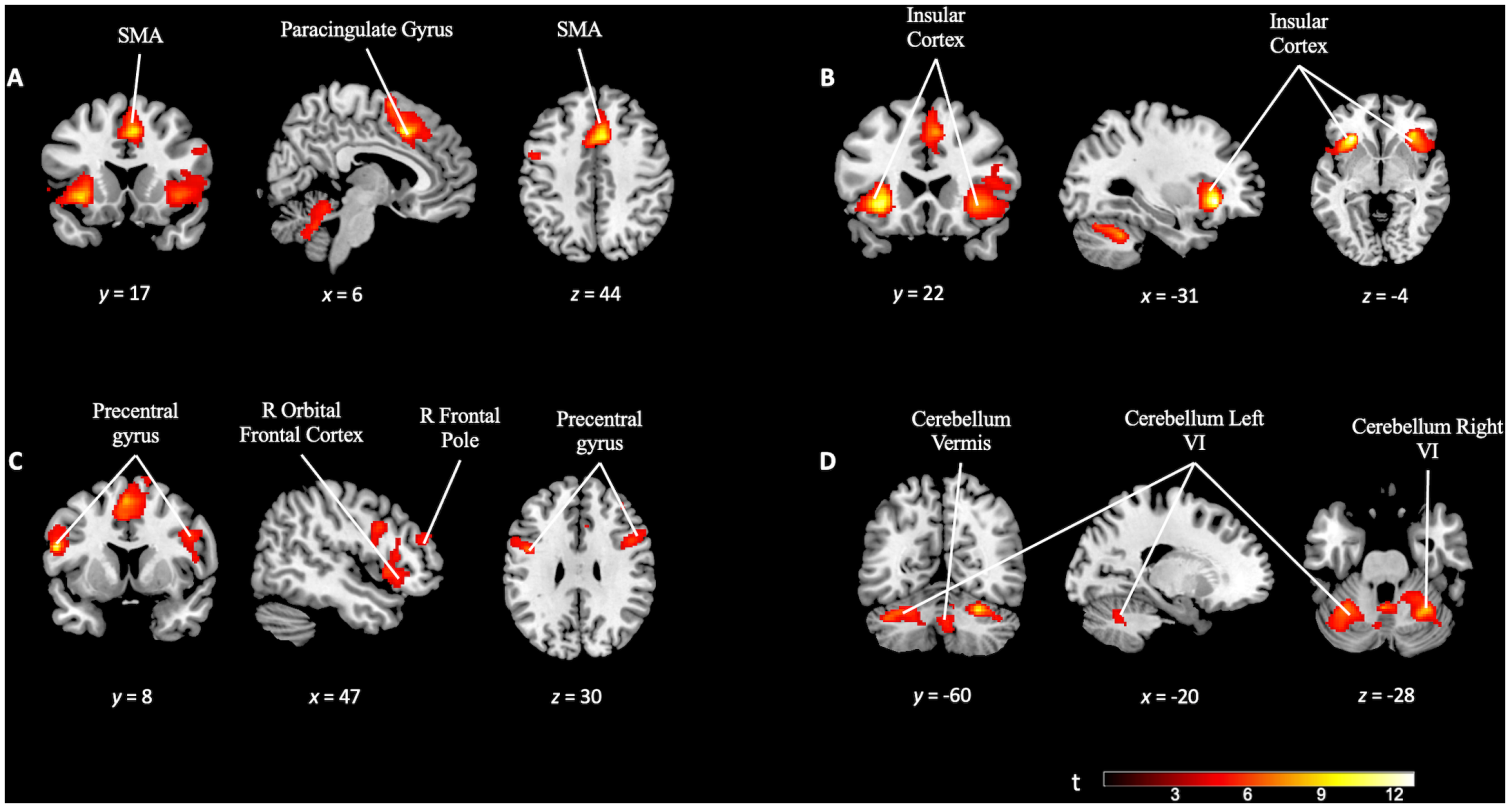
Brain activation pattern of *TD task* contrast (TD task > TD control; FWE cluster-corrected *p* = 0.05; cluster-defining *p* = 0.001; *k* = 328). **(A)** Activation of the paracingulate gyrus and supplementary motor area (SMA). **(B)** The insular cortex, bilaterally, involved in the same TD task. **(C)** Increased activation in the right prefrontal areas (right frontal pole, right orbital frontal cortex) and bilaterally (precentral gyrus). **(D)** Activation pattern in the cerebellum. For more detailed information, see Supplementary Table S1.

Upon examining the *TD task* contrast (TD task > TD control), we were able to study the activation relative to performing the discriminatory task (TD task) compared to non-discriminatory activity (TD control). We applied a family-wise error (FWE) correction at the cluster level at a significance level of *p* = 0.05, with a cluster-defining threshold set at *p* = 0.001, which resulted in a minimum cluster size of 328 voxels (*k* = 328). We observed significant activation in several frontal cortical regions. Specifically, the right frontal orbital cortex, inferior frontal gyrus, superior frontal gyrus, and paracingulate gyrus displayed pronounced engagement during the TD task. Additionally, other areas such as the insular cortex and the cerebellum exhibited notable activity, suggesting their contribution in task execution (see Figure 4 and Supplementary Table S1).

**Figure 5:**
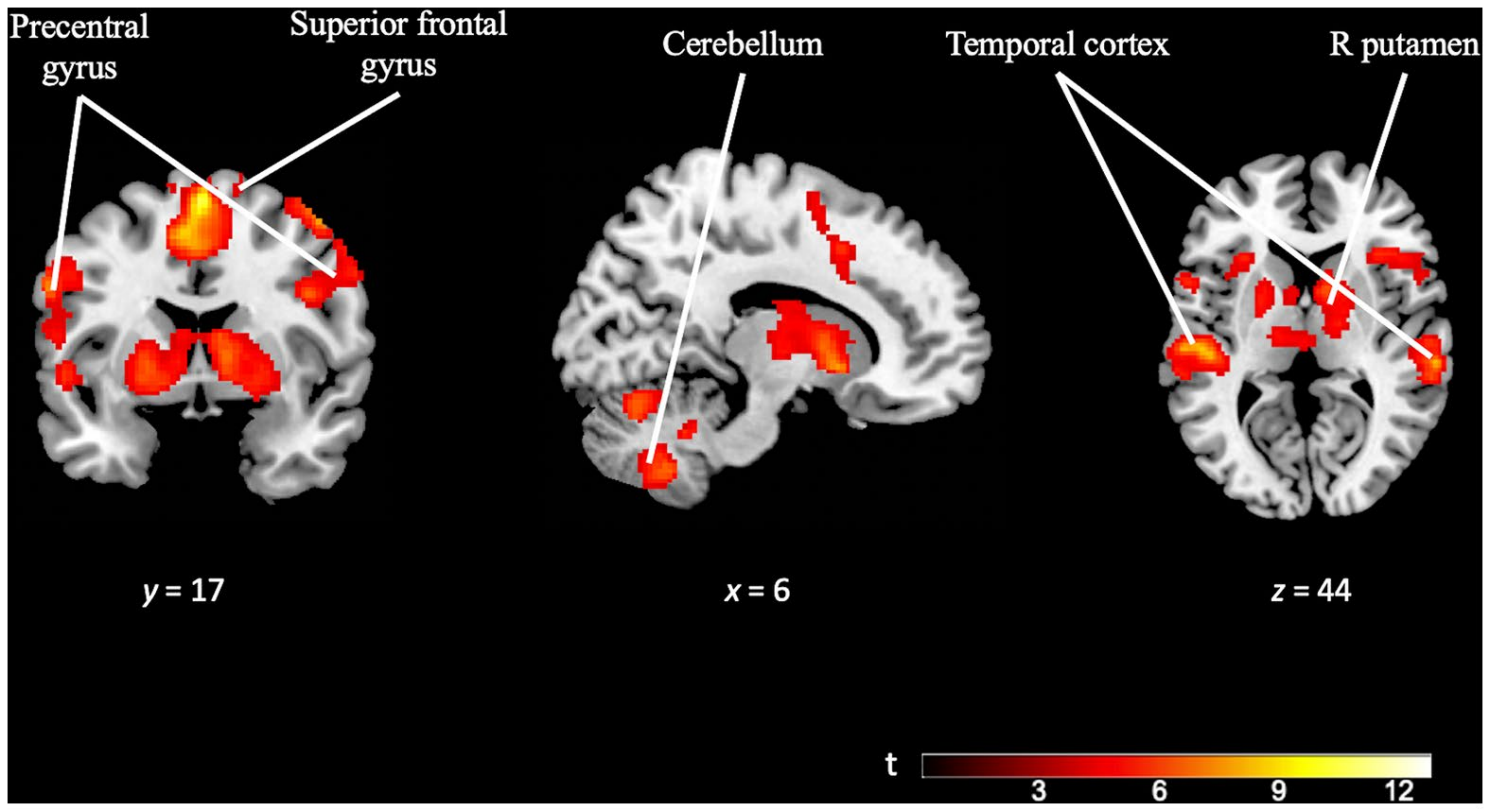
Brain activation pattern of *OD task* contrast (OD task > OD control; FWE cluster-corrected *p* = 0.05; cluster-defining *p* = 0.001; *k* = 402). Image showing significant activation in superior frontal gyrus, precentral gyrus, right putamen, temporal cortex (bilaterally) and cerebellum (bilaterally). For more detailed information on activation areas, see Supplementary Table S2.

Meanwhile, the *OD task* contrast (OD task > OD control) was subjected to FWE cluster correction with a significance threshold of *p* = 0.05. The cluster-defining threshold was set at *p* = 0.001, resulting in a minimum cluster size (*k*) of 402 voxels. This analysis revealed extensive activation across various brain regions. Increased activity was observed in prefrontal areas, including the supplementary motor area (SMA) and the anterior cingulate cortex. In opposition to the TD task contrast, we observed significant activation in subcortical areas, such as the bilateral putamen. Notably, the cerebellum and posterior regions, such as the precuneus, also showed increased activity. Moreover, there was widespread activation in the bilateral temporal cortex (see Figure 5 and Supplementary Table S2).

### 3.2. TD and OD specificity

**Figure 6:**
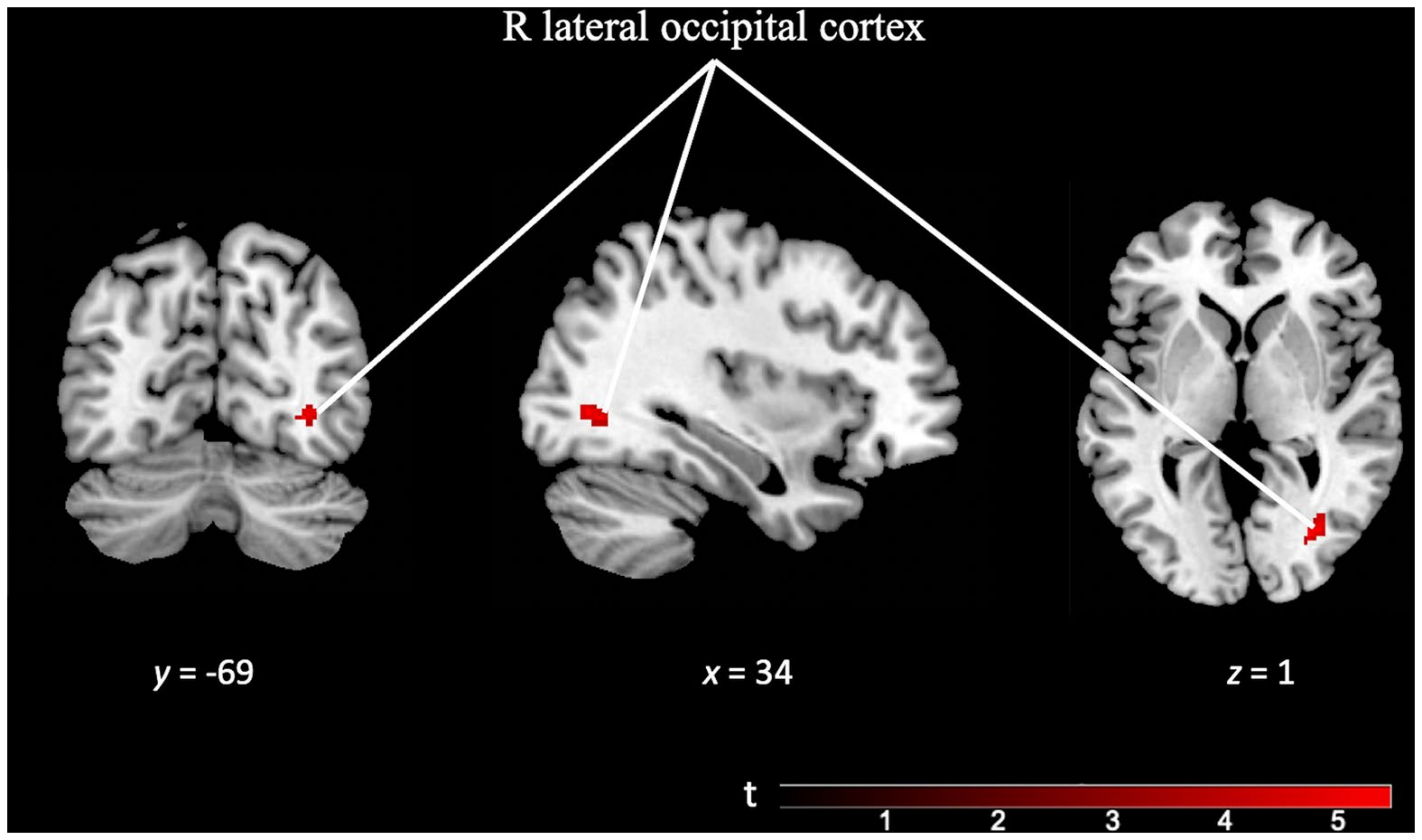
Brain activation pattern of *TD specificity* contrast (TD Task > OD Task; FWE cluster-corrected *p* = 0.05; cluster-defining *p* = 0.001; *k* = 50 vox) showing greater activity in right lateral occipital cortex (see Supplementary Table S3).

After analyzing the contrasts for each paradigm, we performed additional analyses to explore the unique and specific aspects of each task. The *TD specificity* contrast map (TD task > OD task), utilizing a minimum cluster size (*k*) of 50 voxels, revealed distinctive activation localized in the right lateral occipital cortex. Interestingly, this region is not typically associated with temporal discrimination (see Figure 6 and Supplementary Table S3).

**Figure 7:**
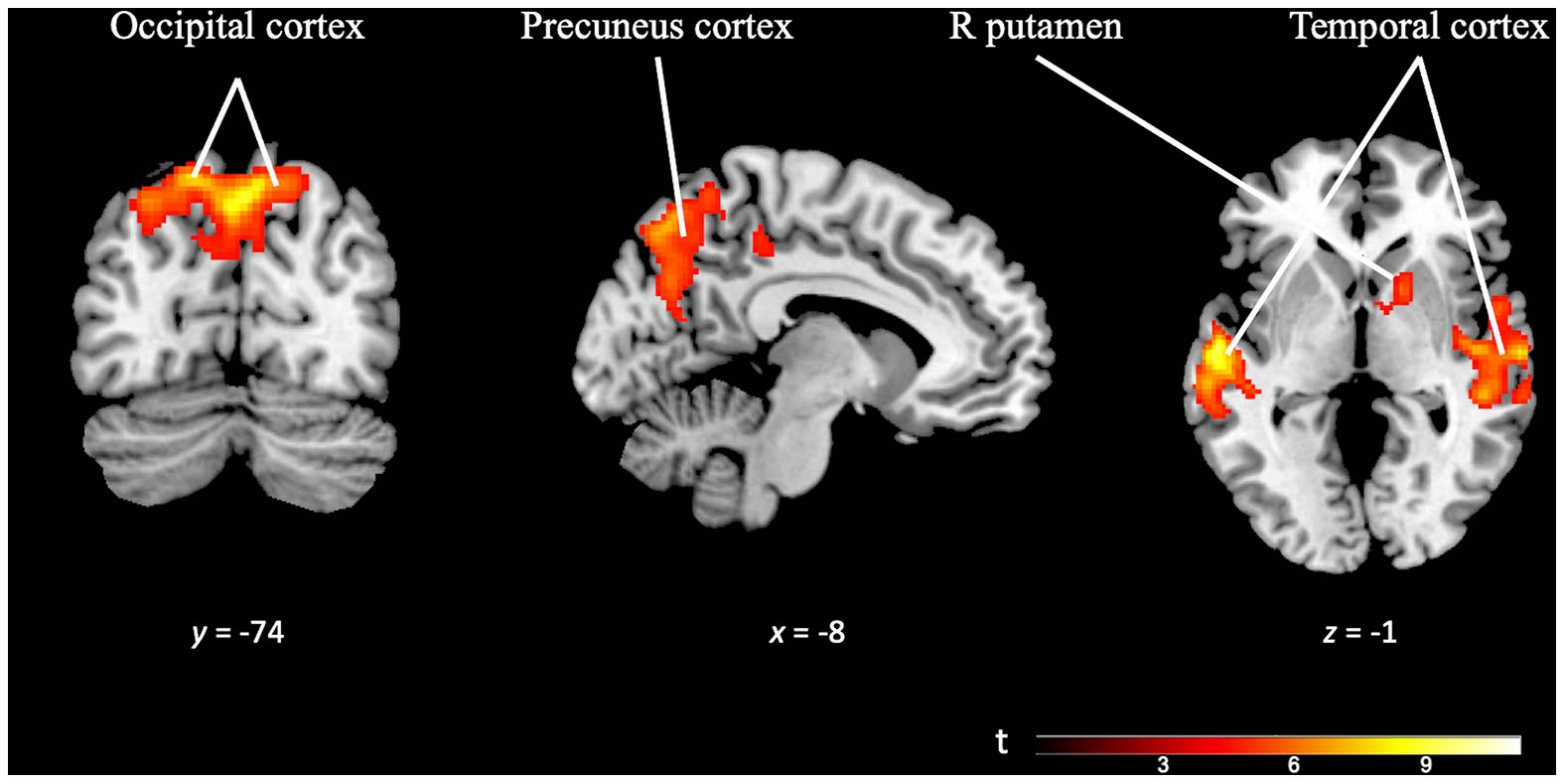
Brain activation pattern of *OD specificity* contrast (OD Task > TD Task; FWE cluster-corrected *p* = 0.05; cluster-defining *p* = 0.001; *k* = 223 vox). The selected slides in this image show greater activity in occipital cortex, precuneus cortex, temporal areas (bilaterally) and right putamen (see Supplementary Table S4).

On the other hand, the *OD specificity* contrast map (OD task > TD task; k = 223 voxels) revealed significant activation across multiple brain regions. The precuneus cortex, temporal areas, pre-supplementary motor area, occipital cortex, and subcortical areas such as the right putamen exhibited prominent activity. Notably, the brain regions recruited by this task were significantly larger than those engaged in the TD task, confirming the differences observed in the separate analyses of each paradigm (see Figure 7 and Supplementary Table S4).

**Figure 8:**
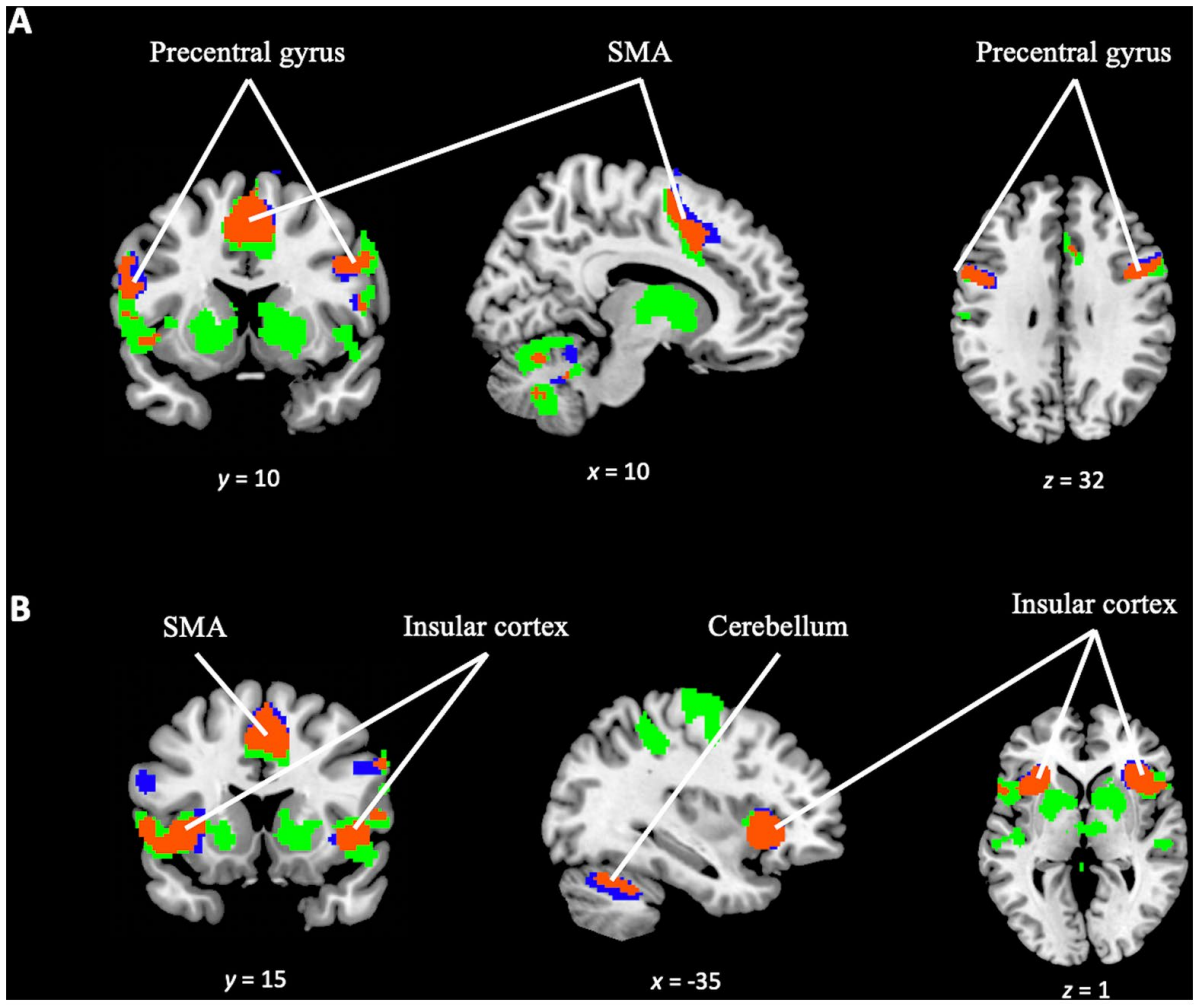
Conjunction analysis between *TD task* and *OD task* contrast. Blue color for *TD task* activation. Green color for *OD task* activation. In orange color, overlapping areas between the two contrasts. **(A)** Overlapping (orange) in supplementary motor area, precentral gyrus (bilaterally and cerebellum). **(B)** Overlapping (orange) in insular cortex (bilaterally), supplementary motor area and cerebellum.

In addition to investigating the specific differences between the tasks, a conjunction analysis was performed between the *TD task* and *OD task* contrast maps. This analysis aimed to examine the overlapping activation and identify common areas of engagement in greater detail. The analysis revealed significant activation overlap in several key regions, including the precentral gyrus, juxtapositional lobule cortex, superior frontal gyrus, paracingulate gyrus, bilateral frontal orbital cortex, bilateral insular cortex, and the cerebellum (see Figure 8). This suggests shared neural substrates and potential interactions between these regions during the execution of both tasks.

### 3.3. Cognitive control

**Figure 9:**
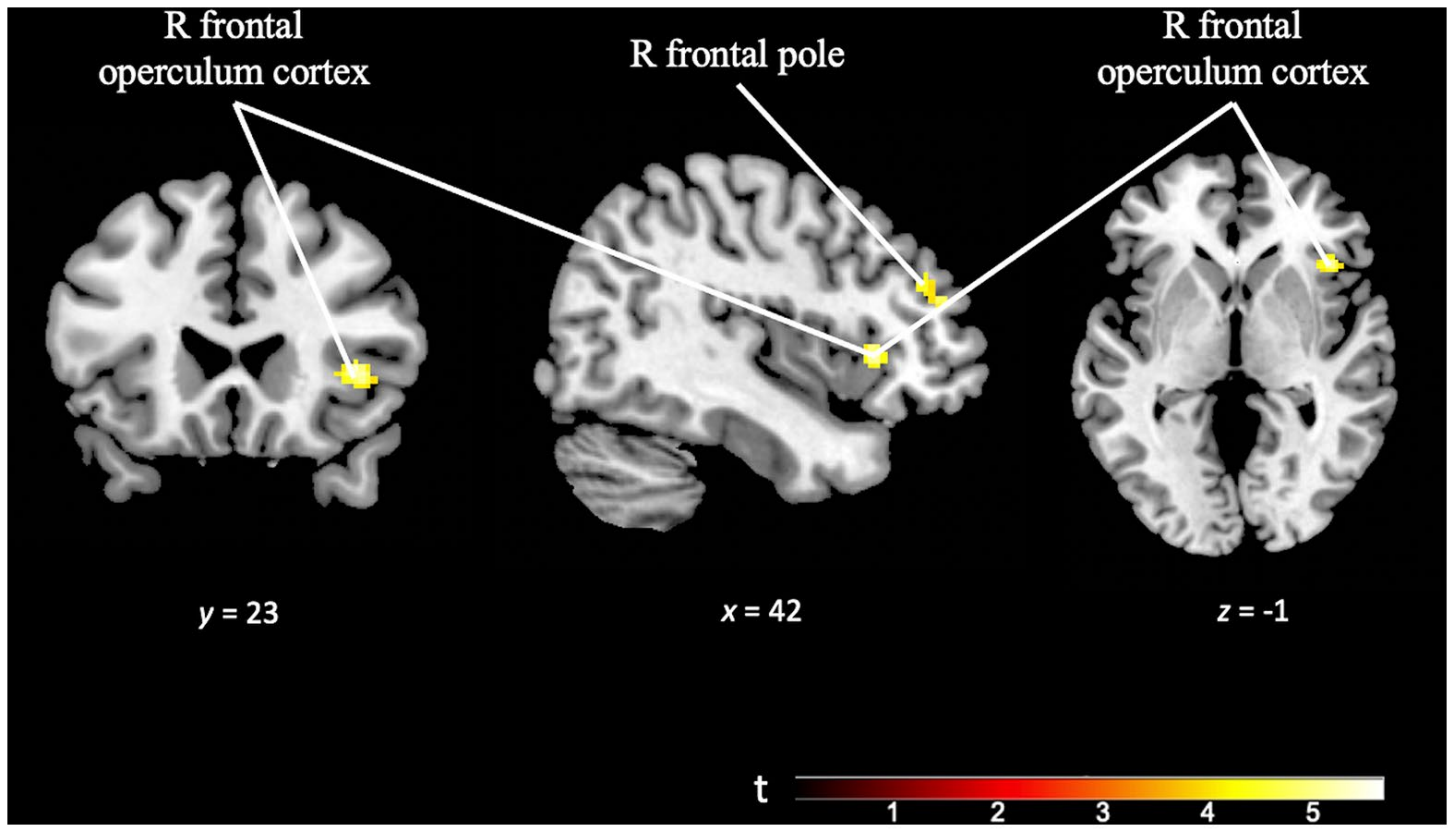
Brain activation pattern of *TD effort* contrast (TD task difficult > TD task easy; *p* = 0.001; *k* = 50 vox) showing greater activation in right frontal pole and right frontal operculum cortex (see Supplementary Table S5).

The component of cognitive effort was examined by analyzing the differences in activation patterns in response to increasing difficulty levels within each paradigm. The *TD effort* contrast (TD Task Difficult > TD Task Easy), employing a minimum cluster size of 50 voxels, revealed activation in right prefrontal areas, specifically the right frontal pole and right frontal operculum. These findings suggest increased cognitive engagement and resource allocation in these regions during the more challenging TD task conditions (see Figure 9 and Supplementary Table S5).

However, the OD paradigm did not reveal discernible differences between various levels of difficulty. When analyzing the *OD effort* contrast (OD Task Difficult > OD Task Easy), no statistically significant differences in the activation patterns were observed. This indicates that the OD task may not induce distinct levels of cognitive effort as detected by the fMRI measurements employed in this study.

**Figure 10:**
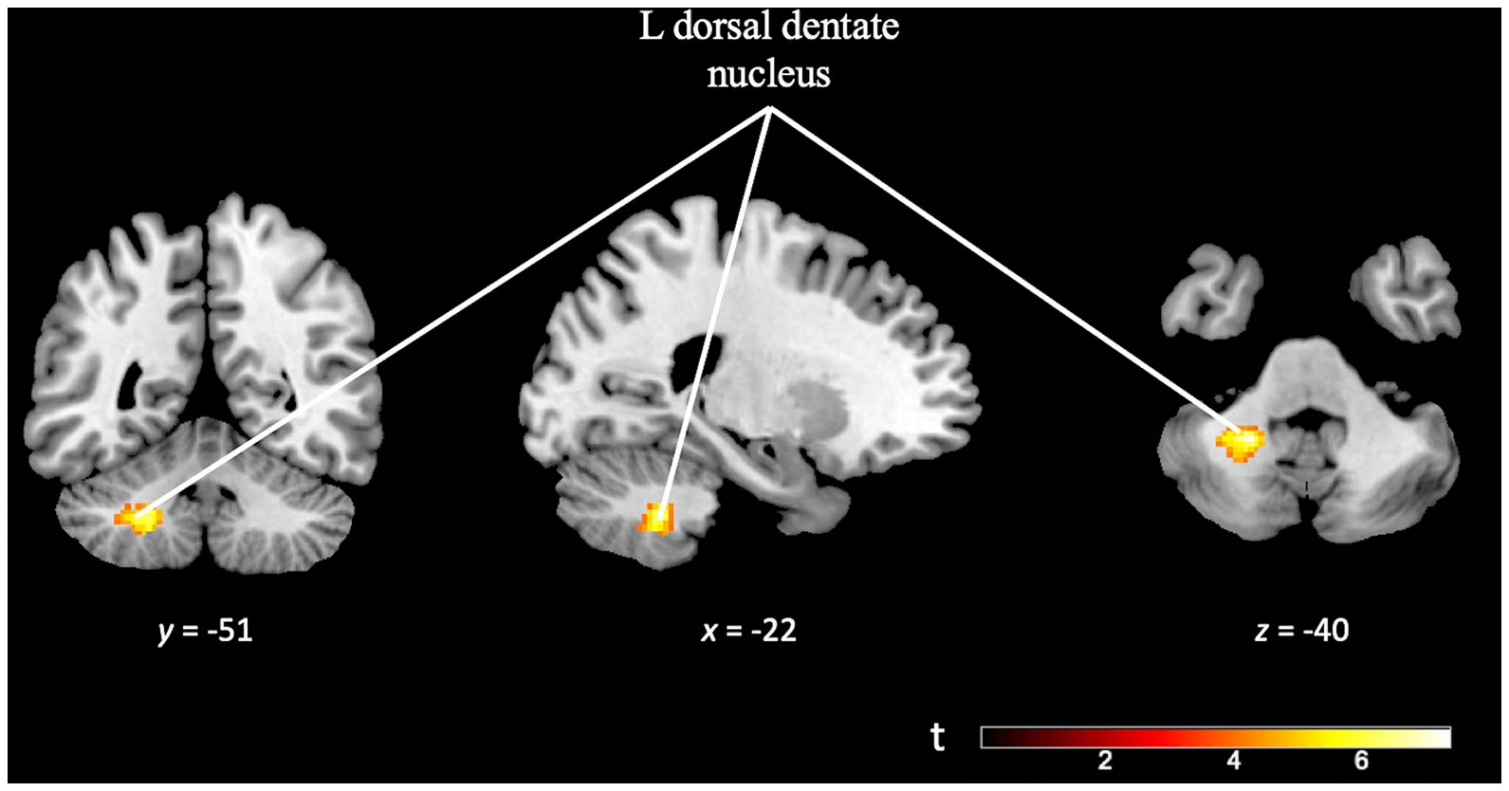
Brain activation pattern of *TD specificity in cognitive effort* contrast, [(TD task difficult > TD task easy) > (OD task difficult > OD task easy)]; FWE cluster-corrected (*p* = 0.05; cluster-defining *p* = 0.001, *k* = 50), showing greater activity in the left dorsal dentate nucleus of the cerebellum (see Supplementary Table S6).

Finally, the *TD specificity in cognitive effort* contrast [(TD task difficult > TD task easy) > (OD task difficult > OD task easy)], minimum cluster size (k) of 50 voxels, revealed activation in the left dorsal dentate nucleus of the cerebellum. This finding suggests that the observed activation in this region is specific to the cognitive effort required during the more challenging TD task (see Figure 10 and Supplementary Table S6).

## 4. Discussion

The findings obtained in this original research contribute to the endeavor to understand time perception. Within the different dimensions encompassed by temporality, in this study we decided to focus on brain activity related to TD in a healthy population. We have attempted to describe the brain regions involved in TD and its interrelation with change detection processes, especially by including a component of higher cognitive load.

Previous studies have pointed out the relationship of temporality with other cognitive processes, such as change detection (Ortuño et al., 2005; Livesey et al., 2007; Alústiza et al., 2018; Garcés et al., 2021). This hypothesized connection becomes more evident when studying processes that require greater cognitive demand, since any cognitive task involves salience and change detection processes (Wiener et al., 2010; Niendam et al., 2012; Ortuño and Alústiza, 2014; Radua et al., 2014; Alústiza et al., 2016, 2017). Therefore, we hypothesize that there is a common brain network underlying time and change detection processes. Following this idea, we conducted a meta-analysis study (Garcés et al., 2021) in which we provided evidence that timing and salience processes are connected. Considering the limitations of a meta-analysis study and in order to corroborate this hypothesis, we considered it worthwhile to perform our own experimental functional neuroimaging experiment in the small-scale proof-of-concept study reported here.

Instead of studying only TD function-specific brain activity, as an isolated and independent brain function, in this experimental study we included tasks for different paradigms (TD and OD) and conditions (cognitive control). This design, based on the interrelation and connection of different brain areas, is closer to the scientific consensus about the real functioning and structure of the brain (de Schotten and Forkel, 2022). Our group has designed and is working on the validation studies of a new experimental computerized perceptual testing tool for TD-OD as a feasible way of studying time perception and its implications. In this proof-of-concept study, we applied a fMRI adaptation of this tool, to examine our hypotheses and evaluate our methodological approach, which has wide applicability in other relevant populations (Kendig, 2015). We consider that the TD process, and the study of the brain circuits involved betters our understanding of cognitive and perceptual processes in the healthy brain and in different neuropsychiatric conditions, especially in schizophrenia (Andreasen et al., 1999; Davalos et al., 2011; Ortuño et al., 2011; Roy et al., 2012; Gómez et al., 2014; Alústiza et al., 2016, 2018; Ciullo et al., 2016; Lošák et al., 2016; Parker et al., 2021; Todd et al., 2022).

### 4.1. TD and OD activity

For each of the two paradigms (TD and OD) we decided to include a *control task*. During this task (passive listening), participants did not have to perform any discriminative activity (passive listening), but only pressed the same button (“1”), regardless of the perceived stimulus (frequent or infrequent). In this way, comparing the *discriminative task* with the *control task*, we were able to assess the evoked activity in each subject in a more detailed way (Sadaghiani et al., 2009; Justen and Herbert, 2018; Kamp et al., 2018).

We describe the pattern of brain activation corresponding to the performance of the TD paradigm. The activation patterns observed (Figure 4) are consistent with those previously reported in the literature (Stevens et al., 2007; Wiener et al., 2010; Ortuño et al., 2011; Nani et al., 2019). In a previous meta-analysis, our research team observed the existence of a cortico-cerebellar-thalamic-cortical circuit, related to time estimation (Ortuño et al., 2011). The same brain areas are the focus of attention for other studies researching temporal processing (Wiener et al., 2010; Matthews and Meck, 2016; Petter et al., 2016; Eichenbaum, 2017; Nobre and van Ede, 2017; Coull and Droit-Volet, 2018; Gili et al., 2018; Nani et al., 2019; Lad et al., 2020; Cona et al., 2021). In conjunction, these findings are consistent with previous evidence for the existence of a brain circuit responsible for temporality.

In this study, we included a non-temporal cognitive task based on the OD paradigm. The brain activity we observed during the performance of OD tasks (greater activity in areas such as the pre-SMA, cerebellum, cingulate cortex and bilateral putamen), was consistent with what is reported in the literature (Ortuño et al., 2005; Stevens et al., 2007; Wiener et al., 2010; Menon, 2015).

### 4.2. Is there a common brain network underlying the TD and OD paradigms?

Given these two contrasts (the *TD activity* and the *OD activity* contrasts) and according to the results of the conjunction analysis between them, we can state that the brain activity corresponding to each of these tasks partially coincides, involving the supplementary motor area, primary motor cortex, prefrontal cortex, insula and cerebellum. However, there are some differences in the activation pattern of both paradigms. In the activity related to the TD paradigm (and not in the *OD task* contrast), we observed greater activity in the right lateral occipital area, in principle, an area not previously described as involved in temporality. In a previous meta-analysis study (Radua et al., 2014) we found a similar finding, although in that study the additional area can be explained by the fact that the task stimuli used there were visual (and not auditory, as they are in this study). In the study, the presence of the additional activation area can plausibly be explained, as follows, in terms of a complication introduced by the test design. While conducting the experiment, both the researchers and participants felt that the TD paradigm was more difficult than the OD paradigm. We suspect that the possible difference in difficulty between tasks (Alexiou et al., 2009) might trigger the recruitment of other brain areas not related to temporality in order to accomplish the TD task successfully. Occipital cortex activation has been described in both auditory and visual oddball tasks (Stevens et al., 2000; Goldman et al., 2009; McDonald et al., 2013; Alústiza et al., 2018; Justen and Herbert, 2018), which would reinforce the idea that it is precisely in tasks that demand greater cognitive effort that network overlap is more evident. Regarding future studies, we believe that it would be convenient to evaluate in a more objective way, not only the performance in execution, but also the difficulty perceived by the participants during the task (Alexiou et al., 2009; Hay et al., 2022).

When we compare the activation pattern for OD with that for TD, we observe that during OD there was greater activation in temporal regions, bilaterally, and in the precuneus. This observation and the differences in general between TD and OD activation patterns may be due to the study design and the ways tasks were presented. In particular, for the TD paradigm tasks, participants had to focus on the duration of the *silence* between two sounds, while for the OD paradigm tasks, participants had to focus on the frequency of a *sound*. So, it is to be expected that brain areas involved in auditory processes would be activated in OD paradigm tasks, as we observed.

Despite the limitations of the study, in view of the fact that the differences between brain activation patterns were small, predictable and also explainable by the limitations of the study design, our results support the hypothesis that the network defined under the TD paradigm has areas in common with other cognitive networks and processes such as OD (Alústiza et al., 2016, 2017, 2018; Garcés et al., 2021).

### 4.3. Cognitive effort

By establishing easy and difficult modes of the two experimental tasks, we aimed to study the effect of cognitive control on activation patterns. Regarding TD, in the *TD effort* contrast we found that increased difficulty resulted in implication of right prefrontal regions (specifically, the right frontal pole and the right frontal operculum cortex), which is suggestive of involvement of the dorsolateral prefrontal cortex (DLPFC), which has been previously described as part of a multi-demand system related to more complex cognitive functions and tasks (Tregellas et al., 2006; Lewandowska et al., 2010; Koizumi et al., 2018; Chen et al., 2020; Hertrich et al., 2021). These findings are consistent with the hypothesis that brain networks invoked in TD are engaged in tasks that require greater cognitive difficulty (Livesey et al., 2007; Davalos et al., 2011; Niendam et al., 2012; Alústiza et al., 2016, 2017, 2018).

The *OD effort* contrast did not show statistically significant differences. This could be because the increase of difficulty in OD task (difference between *OD difficult* and *OD easy*) is perhaps not demanding enough to activate the regions involved in greater cognitive effort. In addition, the small sample size in this preliminary study makes it difficult to obtain robust results.

Is there any brain region specific to TD, not active during OD, whose role becomes more important with increasing cognitive load? Interestingly, comparison of the contrast map for increase in difficulty in the TD task with the contrast map for increase in difficulty in the OD task, reveals greater activation in cerebellar regions (see Figure 10). Numerous studies have pointed out the relevant role of the cerebellum in temporal processing and its relationship with areas such as the SMA, parietal cortex, thalamus and basal ganglia (Bengtsson et al., 2005; Koziol et al., 2014; Finnerty et al., 2015; Petter et al., 2016; Bareš et al., 2019). Particularly, detection of the involvement of the cerebellum and subcortical areas in temporal processing is clearer when using tasks of sub-second timing conditions, as in the present study (Petter et al., 2016; Nani et al., 2019).

The pattern of cerebellar activity detected has been reported to be related to motor functions and complex cognitive functions, especially those that involve prefrontal areas (Bernard et al., 2014). This result points at the possibility of the TD network not only being involved in time perception processes, but also being relevant and perhaps playing a specific mediating role in more complex cognitive processes (such as executive functions) (Niendam et al., 2012; Radua et al., 2014; Lošák et al., 2016; Bareš et al., 2019; Pinheiro et al., 2021).

### 4.4. Limitations

Some of the limitations of this study – the modest sample size, in particular – are inherent to a proof-of-concept study (Kendig, 2015). The proposed theoretical framework of the brain circuits involved in TD processes and their implication in other cognitive functions, being derived from a small sample size, must be taken with caution. Nevertheless, we believe that the insights gained in our experimental study are applicable to larger samples and clinical populations for future research oriented to the implementation and validation of our test-tool. Despite these limitations, the present results are consistent with previous evidence from other task-based neuroimaging studies (Wiener et al., 2010; Niendam et al., 2012; Nani et al., 2019; Garcés et al., 2021), which may help promote larger scale studies.

Another limitation of our study is the age range of participants. To increase sample size and generalizability to the population, we included healthy subjects aged 18 to 65 in our study protocol. In the final sample of participants, the age range was between 23 and 49 years old. However, it is important to note that age-related changes in time discrimination can affect both test performance and neuroimaging results in older subjects (Mioni et al., 2021). In future studies examining temporality and other cognitive functions, it would be beneficial to consider the impact of participant age on the findings. A possible solution could be to include younger subjects, or to expand the sample size to study the effect of age in more detail as a possible confounding factor. An alternative approach would be to compare the findings with performance in other domains (such as processing speed), which have been known to be influenced by age (Baudouin et al., 2018).

In this study, the nature of the tests implied certain limitations: some of the differences in the TD and OD task activation patterns can be attributed to the greater auditory component of the latter task; it is possible that TD task was more difficult to execute than the OD task; and it is hard to determine accurately the increased cognitive load between the two levels of the OD task. We believe, however, that such limitations have not been entirely detrimental but also provided us with valuable information on brain functioning.

The general heterogeneity in neuroimaging research is a big concern. Although this heterogeneity is partly attributable to the range of experimental task employed, a priority in our opinion is to describe differences between individual subjects. To do this, there is a need for further fMRI studies involving more participants and participants with different neuropsychiatric conditions.

Despite recent improvements in the field of neuroimaging, each of the available imaging techniques provides us with a partial view of the real brain structure and function. Integrating different techniques and associating them with fMRI studies can be valuable to study primary domains such as TD. Having looked at the relationship between time perception and change detection, it would be interesting to study the evoked potentials, such as, mismatch negativity (MMN) (Moore et al., 2021). The combinations of findings from task-based fMRI studies and studies that focus on brain structure (i.e., diffusion techniques) may help us to better understand brain connectivity and functioning (de Schotten and Forkel, 2022) especially in disorders such as schizophrenia where aberrant temporal processing has been described (Alústiza et al., 2016, 2017, 2018; Ciullo et al., 2016) and may be related to abnormal structural brain connectivity (white matter impairment) (Ortuño et al., 2011; Gómez et al., 2014; Kochunov et al., 2017, 2022).

## 5. Conclusion

Our study contributes to knowledge about the brain network responsible for temporal perception. In particular, we were interested in the brain areas related to TD and their interrelation with cognitive processes involving change detection in the healthy population. To that end, based on the TD and OD paradigms, we designed a test-tool including a cognitive control component and adapted it for use within MRI equipment. Our results indicate the existence of a relationship between the brain circuits of TD and other discriminative cognitive processes (OD), and in addition shed some light on how these TD circuits could participate in more complex cognitive processes (i.e., executive functions). We provide experimental proof-of-concept evidence, consistent with findings in previous studies, that supports our hypotheses and validates the methodology for application in larger populations. We consider this to be important for research on various neuropsychiatric conditions and, especially, schizophrenia.

## Data availability statement

The original contributions presented in the study are included in the article/Supplementary material, further inquiries can be directed to the corresponding author.

## Ethics statement

The studies involving human participants were reviewed and approved by Research Ethics Committee of the University of Navarra. The patients/participants provided their written informed consent to participate in this study.

## Author contributions

FO conceived the idea of the experiment and the original hypothesis. MF-S designed the original experimental measurement tool. JG, IA, MF-S, MF, and FO worked on the study design and adapted the measurement tool to MRI. JG and CV-A were involved in the recruitment and initial screening interview of participants. JG led the experiment, under the supervision of and with the help of CV-A and IA. RG-E was responsible for the diagnosis and clinical reporting of structural brain imaging findings. MF-S was responsible for the pre-processing of the fMRI images. MF-S and JG performed the statistical analysis of the results and developed the images. JG wrote the manuscript. IA, CV-A, PM, MF-S, and FO commented, corrected and approved the manuscript. All authors contributed to the article and approved the submitted version.

## Funding

This research has been financed by a project of the Carlos III Health Institute. Health Institutions (Project File Number: PI17/00240). Funded by *Instituto de Salud Carlos III* and co-funded by European Union (ERDF/ESF, “A way to make Europe”).

## Conflict of interest

PM is supported by Clínica Universidad de Navarra and has received research grants from the Ministry of Education (Spain), the Government of Navarra (Spain) and the Spanish Foundation of Psychiatry and Mental Health and AstraZeneca; he is a clinical consultant for MedAvante-ProPhase and has received lecture honoraria from and/or has been a consultant for AB-Biotics, Adept Field Solutions, Guidepoint, Janssen, Novumed, Roland Berger and Scienta.

The remaining authors declare that the research was conducted in the absence of any commercial or financial relationships that could be construed as a potential conflict of interest.

## Publisher’s note

All claims expressed in this article are solely those of the authors and do not necessarily represent those of their affiliated organizations, or those of the publisher, the editors and the reviewers. Any product that may be evaluated in this article, or claim that may be made by its manufacturer, is not guaranteed or endorsed by the publisher.

## Abbreviations

fMRI: functional magnetic resonance imaging
ISI: inter-stimulus interval
TD: time discrimination
OD: oddball detection
