# Supplementary material for: Time discrimination and change detection could share a common brain network: findings of a task-based fMRI study
Source: Front Psychol. 2023 Jun 22;14:1110972. doi: 10.3389/fpsyg.2023.1110972 (PMC10390230; doi:10.3389/fpsyg.2023.1110972)
Supplement: Supplementary file 1 [file Table_1.DOCX]

Supplementary Material

**Supplementary Table 1**. *TD task* contrast (TD task > TD control; FWE cluster-corrected p = 0.05; cluster-defining p=0.001; *k*=328).

| **Cluster** | **Cluster Size** | **Cluster-level p value (FWE-corrected)** | **Local Maxima** | | | | |
| --- | --- | --- | --- | --- | --- | --- | --- |
|  |  |  | **MNI coordinates** | | | **T-value** | **Anatomic Region** |
|  |  |  | **X** | **Y** | **Z** |  |  |
| 1 | 1528 vox | < 0.001 | +4 | +16 | +42 | 10.3 | Paracingulate Gyrus |
|  |  |  | +2 | +4 | +66 | 8.59 | Juxtapositional Lobule Cortex |
|  |  |  | -2 | +8 | +56 | 8.09 | Juxtapositional Lobule Cortex |
|  |  |  | +4 | +34 | +42 | 4.89 | Superior Frontal Gyrus |
|  |  |  | +12 | +6 | +76 | 4.36 | Superior Frontal Gyrus |
| 2 | 1438 vox | < 0.001 | +34 | +30 | -4 | 9.24 | Frontal Orbital Cortex |
|  |  |  | +42 | +14 | -2 | 6.53 | Insular Cortex |
|  |  |  | +46 | +10 | +30 | 5.83 | Precentral Gyrus |
|  |  |  | +46 | +22 | +10 | 5.16 | Inferior Frontal Gyrus, pars triangularis |
|  |  |  | +56 | +14 | +32 | 5.13 | Precentral Gyrus |
|  |  |  | +50 | +8 | +14 | 4.75 | Inferior Frontal Gyrus, pars opercularis |
|  |  |  | +46 | +4 | +20 | 4.65 | Precentral Gyrus |
|  |  |  | +48 | +6 | +16 | 4.64 | Inferior Frontal Gyrus, pars opercularis |
|  |  |  | +54 | +12 | +10 | 4.39 | Inferior Frontal Gyrus, pars opercularis |
|  |  |  | +50 | +24 | +22 | 4.38 | Inferior Frontal Gyrus, pars opercularis |
| 3 | 1385 vox | < 0.001 | +26 | -60 | -26 | 10 | Cerebellum Right VI |
|  |  |  | -2 | -54 | -28 | 7.39 | Cerebellum Left I-IV |
|  |  |  | -6 | -68 | -32 | 6.04 | Cerebellum Vermis VIIb |
|  |  |  | +4 | -46 | -16 | 5.73 | Cerebellum Right I-IV |
|  |  |  | +4 | -50 | -18 | 5.6 | Cerebellum Right I-IV |
|  |  |  | +4 | -60 | -38 | 5.32 | Cerebellum Vermis VIIb |
|  |  |  | +40 | -60 | -32 | 5.03 | Cerebellum Right Crus I |
|  |  |  | +14 | -44 | -28 | 4.98 | Cerebellum Right V |
|  |  |  | +14 | -48 | -28 | 4.76 | Cerebellum Right V |
|  |  |  | +16 | -54 | -34 | 4.64 | Cerebellum Right VI |
| 4 | 876 | < 0.001 | -30 | +26 | -4 | 12.7 | Frontal Orbital Cortex |
|  |  |  | -56 | +14 | +0 | 4.62 | Inferior Frontal Gyrus, pars opercularis |
|  |  |  | -54 | +12 | +6 | 4.54 | Inferior Frontal Gyrus, pars opercularis |
|  |  |  | -31 | +22 | -4 | 4.50 | Insular Cortex |
| 5 | 729 | < 0.001 | -30 | -50 | -32 | 7.85 | Cerebellum Left VI |
|  |  |  | -32 | -56 | -28 | 6.84 | Cerebellum Left VI |
|  |  |  | -38 | -56 | -30 | 6.52 | Cerebellum Left Crus I |
|  |  |  | -32 | -66 | -26 | 6.35 | Cerebellum Left VI |
|  |  |  | -30 | -68 | -24 | 6.12 | Cerebellum Left VI |
|  |  |  | -16 | -52 | -40 | 5.14 | Dorsal Dentate left Nucleus |
| 6 | 623 | 0.002 | -56 | +10 | +22 | 12.5 | Inferior Frontal Gyrus, pars opercularis |
|  |  |  | -46 | +2 | +32 | 7.67 | Precentral Gyrus |
|  |  |  | -46 | +2 | +38 | 6.95 | Precentral Gyrus |
|  |  |  | -52 | 0 | +50 | 5.65 | Precentral Gyrus |
|  |  |  | -46 | -4 | +58 | 5.1 | Precentral Gyrus |
| 7 | 328 | 0.037 | +40 | +40 | +24 | 6.97 | Frontal Pole |

**Supplementary Table 2**. *OD task* contrast (OD task > OD control; FWE cluster-corrected p = 0.05; cluster-defining p=0.001; *k*=402).

| **Cluster** | **Cluster Size** | **Cluster-level p value (FWE-corrected)** | **Local Maxima** | | | | |
| --- | --- | --- | --- | --- | --- | --- | --- |
|  |  |  | **MNI coordinates** | | | **T-value** | **Anatomic Region** |
|  |  |  | **X** | **Y** | **Z** |  |  |
| 1 | 7397 vox | < 0.001 | -44 | -6 | +60 | 11.5 | Precentral Gyrus |
|  |  |  | -40 | -38 | +50 | 8.52 | Postcentral Gyrus |
|  |  |  | -38 | -14 | +68 | 8.38 | Precentral Gyrus |
|  |  |  | -30 | -6 | +48 | 8.1 | Precentral Gyrus |
|  |  |  | +14 | +12 | -4 | 7.61 | Right Putamen |
|  |  |  | -48 | -30 | +60 | 7.54 | Postcentral Gyrus |
|  |  |  | -46 | -6 | +48 | 7.32 | Precentral Gyrus |
|  |  |  | -60 | +8 | +30 | 7.24 | Precentral Gyrus |
|  |  |  | -24 | -2 | +72 | 7.22 | Superior Frontal Gyrus |
|  |  |  | -52 | +10 | -4 | 7.14 | Temporal Pole |
|  |  |  | -52 | +10 | -4 | 7.14 | Temporal Pole |
|  |  |  | -28 | +22 | +2 | 6.37 | Insular Cortex |
|  |  |  | -30 | +24 | -4 | 6.14 | Insular Cortex |
|  |  |  | -56 | +14 | +2 | 6.12 | Inferior Frontal Gyrus, pars opercularis |
|  |  |  | -38 | +16 | -6 | 5.99 | Insular Cortex |
|  |  |  | -48 | 14 | -8 | 5.86 | Temporal Pole |
| 2 | 2856 vox | < 0.001 | +24 | -60 | -24 | 10.6 | Cerebellum Right VI |
|  |  |  | +20 | -64 | -18 | 9.11 | Cerebellum Right VI |
|  |  |  | +18 | -58 | -48 | 7.95 | Cerebellum Right VIIIb |
|  |  |  | +6 | -60 | -40 | 7.41 | Cerebellum Right IX |
|  |  |  | +8 | -72 | -26 | 6.75 | Cerebellum Right VI |
|  |  |  | +38 | -62 | -28 | 6.5 | Cerebellum Right Crus I |
|  |  |  | +10 | -70 | -24 | 6.34 | Cerebellum Right VI |
|  |  |  | +0 | -50 | -24 | 6.02 | Cerebellum Right I-IV |
|  |  |  | +2 | -54 | -24 | 5.9 | Cerebellum Right I-IV |
|  |  |  | +8 | -48 | -26 | 5.86 | Cerebellum Right I-IV |
| 3 | 1647 vox | < 0.001 | +0 | +0 | +66 | 10.9 | Juxtapositional Lobule Cortex |
|  |  |  | -4 | +4 | +52 | 8.63 | Juxtapositional Lobule Cortex |
|  |  |  | +6 | +12 | +44 | 8.62 | Paracingulate Gyrus |
|  |  |  | +0 | +8 | +46 | 7.45 | Paracingulate Gyrus |
|  |  |  | +2 | +26 | +30 | 4.46 | Cingulate Gyrus, anterior division |
|  |  |  | +8 | +22 | +28 | 4.24 | Cingulate Gyrus, anterior division |
|  |  |  | -12 | +6 | +70 | 4.24 | Superior Frontal Gyrus |
|  |  |  | +16 | +6 | +70 | 4.15 | Superior Frontal Gyrus |
| 4 | 1204 vox | < 0.001 | -64 | -38 | +16 | 9.17 | Planum Temporale |
|  |  |  | -48 | -24 | +6 | 8.84 | Heschl´s Gyrus |
|  |  |  | -56 | -22 | +6 | 8.21 | Planum Temporale |
|  |  |  | -50 | -36 | +14 | 6.89 | Planum Temporale |
|  |  |  | -56 | -18 | +28 | 5.53 | Postcentral Gyrus |
|  |  |  | -62 | -18 | +16 | 4.92 | Central Opercular Cortex |
|  |  |  | -64 | -18 | +20 | 4.85 | Postcentral Gyrus |
|  |  |  | -40 | -36 | +12 | 4.6 | Planum Temporale |
|  |  |  | -60 | -18 | +44 | 4.14 | Postcentral Gyrus |
|  |  |  | -58 | -20 | +40 | 4.12 | Postcentral Gyrus |
| 5 | 743 vox | < 0.001 | +62 | -28 | +8 | 7.86 | Superior Temporal Gyrus, posterior division |
|  |  |  | +64 | -36 | +14 | 7.61 | Supramarginal Gyrus, posterior division |
|  |  |  | +56 | -30 | +12 | 7.3 | Planum Temporale |
|  |  |  | +60 | -18 | +10 | 6.26 | Planum Temporale |
|  |  |  | +66 | -36 | +24 | 6.16 | Supramarginal Gyrus, posterior division |
|  |  |  | +56 | -24 | -4 | 5.66 | Superior Temporal Gyrus, posterior division |
|  |  |  | +54 | -22 | +0 | 5.46 | Superior Temporal Gyrus, posterior division |
|  |  |  | +52 | -34 | +18 | 5.03 | Planum Temporale |
| 6 | 715 vox | < 0.001 | +38 | +2 | +62 | 13.5 | Middle Frontal Gyrus |
|  |  |  | +46 | +4 | +56 | 10.4 | Middle Frontal Gyrus |
|  |  |  | +60 | +10 | +36 | 9.16 | Precentral Gyrus |
|  |  |  | +42 | +6 | +28 | 6.32 | Precentral Gyrus |
|  |  |  | +44 | +8 | +30 | 6.12 | Precentral Gyrus |
|  |  |  | +56 | +8 | +44 | 5.67 | Precentral Gyrus |
|  |  |  | +36 | -6 | +68 | 4.92 | Precentral Gyrus |
|  |  |  | +28 | -4 | +52 | 4.76 | Middle Frontal Gyrus |
|  |  |  | +32 | -2 | +48 | 4.57 | Middle Frontal Gyrus |
| 7 | 413 | < 0.001 | +44 | +22 | -4 | 7.11 | Frontal Operculum Cortex |
|  |  |  | +42 | +14 | 0.0 | 7.06 | Insular Cortex |
|  |  |  | +36 | +26 | +4 | 6.68 | Frontal Operculum Cortex |
|  |  |  | +34 | +28 | -4 | 6.66 | Frontal Orbital Cortex |
| 8 | 402 vox | 0.004 | -20 | -62 | -24 | 5.97 | Cerebellum Left VI |
|  |  |  | -28 | -48 | -30 | 5.56 | Cerebellum Left VI |
|  |  |  | -40 | -62 | -30 | 5.37 | Cerebellum Left Crus I |
|  |  |  | -36 | -62 | -28 | 5.23 | Cerebellum Left Crus I |

**Supplementary Table 3**. *TD specificity* contrast (TD Task > OD Task; FWE cluster-corrected p = 0.05; cluster-defining p=0.001; *k*=50 vox).

| **Cluster** | **Cluster Size** | **Cluster-level p value (FWE-corrected)** | **Local Maxima** | | | | |
| --- | --- | --- | --- | --- | --- | --- | --- |
|  |  |  | **MNI coordinates** | | | **T-value** | **Anatomic Region** |
|  |  |  | **X** | **Y** | **Z** |  |  |
| 1 | 51 vox | 0.745 | +32 | -72 | +0 | 5.42 | Lateral Occipital Cortex, inferior division |

**Supplementary Table 4**. *OD specificity* contrast (OD Task > TD Task; FWE cluster-corrected p = 0.05; cluster-defining p=0.001; *k*=223 vox).

| **Cluster** | **Cluster Size** | **Cluster-level p value (FWE-corrected)** | **Local Maxima** | | | | |
| --- | --- | --- | --- | --- | --- | --- | --- |
|  |  |  | **MNI coordinates** | | | **T-value** | **Anatomic Region** |
|  |  |  | **X** | **Y** | **Z** |  |  |
| 1 | 4301 vox | < 0.001 | +6 | -72 | +48 | 8.59 | Precuneous Cortex |
|  |  |  | -16 | -72 | +54 | 7.55 | Lateral Occipital Cortex, superior division |
|  |  |  | -4 | -68 | +52 | 6.62 | Precuneous Cortex |
|  |  |  | -30 | -70 | +46 | 6.25 | Lateral Occipital Cortex, superior division |
|  |  |  | +6 | -68 | +34 | 6.04 | Precuneous Cortex |
|  |  |  | -26 | -64 | +52 | 5.99 | Lateral Occipital Cortex, superior division |
|  |  |  | -30 | -64 | +42 | 5.99 | Lateral Occipital Cortex, superior division |
|  |  |  | -12 | -68 | +24 | 5.94 | Precuneous Cortex |
|  |  |  | -12 | -70 | +32 | 5.89 | Precuneous Cortex |
|  |  |  | -46 | -36 | +52 | 5.7 | Postcentral Gyrus |
| 2 | 3128 vox | < 0.001 | +54 | -18 | +4 | 9.57 | Planum Temporale |
|  |  |  | +46 | -12 | -14 | 9.49 | Superior Temporal Gyrus, posterior division |
|  |  |  | +56 | -16 | +6 | 9.32 | Planum Temporale |
|  |  |  | +52 | -2 | -14 | 8.78 | Superior Temporal Gyrus, anterior division |
|  |  |  | +52 | -24 | -8 | 7.92 | Middle Temporal Gyrus, posterior division |
|  |  |  | +56 | -24 | -6 | 7.84 | Middle Temporal Gyrus, posterior division |
|  |  |  | +52 | -10 | -10 | 7.72 | Superior Temporal Gyrus, posterior division |
|  |  |  | +52 | -6 | -8 | 7.72 | Superior Temporal Gyrus, anterior division |
|  |  |  | +52 | -18 | -8 | 7.59 | Middle Temporal Gyrus, posterior division |
|  |  |  | +54 | +0 | -10 | 7.55 | Superior Temporal Gyrus, anterior division |
| 3 | 2156 vox | < 0.001 | -58 | -12 | +4 | 11.1 | Planum Temporale |
|  |  |  | -62 | -22 | +6 | 10.8 | Planum Temporale |
|  |  |  | -50 | -26 | -12 | 8.58 | Middle Temporal Gyrus, posterior division |
|  |  |  | -56 | -8 | -8 | 8.46 | Superior Temporal Gyrus, anterior division |
|  |  |  | -58 | -38 | +10 | 7.75 | Superior Temporal Gyrus, posterior division |
|  |  |  | -56 | -24 | -8 | 6.97 | Middle Temporal Gyrus, posterior division |
|  |  |  | -60 | -36 | +14 | 6.96 | Planum Temporale |
|  |  |  | -50 | -22 | +4 | 6.87 | Heschl´s Gyrus |
|  |  |  | -56 | -14 | -12 | 6.81 | Middle Temporal Gyrus, posterior division |
|  |  |  | -62 | -24 | +18 | 6.68 | Postcentral Gyrus |
| 4 | 311 vox | 0.013 | -38 | +56 | +4 | 7.48 | Frontal Pole |
|  |  |  | -34 | +60 | +6 | 6.22 | Frontal Pole |
|  |  |  | -32 | +58 | +10 | 6.15 | Frontal Pole |
|  |  |  | -48 | +32 | +34 | 5.71 | Middle Frontal Gyrus |
|  |  |  | -38 | +42 | +32 | 4.98 | Frontal Pole |
|  |  |  | -36 | +34 | +38 | 4.86 | Middle Frontal Gyrus |
|  |  |  | -34 | +50 | +20 | 4.46 | Frontal Pole |
|  |  |  | -22 | +64 | +8 | 4.43 | Frontal Pole |
|  |  |  | -38 | +46 | +24 | 4.18 | Frontal Pole |
|  |  |  | -40 | +52 | +20 | 4.04 | Frontal Pole |
| 5 | 241 vox | 0.037 | +2 | +0 | +46 | 5.16 | Cingulate Gyrus, anterior division |
| 6 | 223 vox | 0.048 | +16 | +10 | +2 | 5.41 | Right Putamen |
|  |  |  | +18 | +16 | -4 | 5.16 | Right Putamen |
|  |  |  | +12 | -10 | +6 | 4.56 | Right Thalamus |
|  |  |  | +14 | -8 | +8 | 4.49 | Right Thalamus |
|  |  |  | +8 | +2 | -2 | 4.44 | Right Putamen |
|  |  |  | +14 | +0 | +6 | 4.41 | Right Pallidum |
|  |  |  | +16 | -2 | +8 | 4.41 | Right Putamen |
|  |  |  | +2 | +4 | -4 | 4.3 | Right Putamen |
|  |  |  | +12 | -10 | +2 | 4.12 | Right Thalamus |

**Supplementary Table 5**. *TD effort* contrast (TD task difficult > TD task easy; p=0.001; *k*=50 vox).

| **Cluster** | **Cluster Size** | **Cluster-level p value (FWE-corrected)** | **Local Maxima** | | | | |
| --- | --- | --- | --- | --- | --- | --- | --- |
|  |  |  | **MNI coordinates** | | | **T-value** | **Anatomic Region** |
|  |  |  | **X** | **Y** | **Z** |  |  |
| 1 | 89 vox | 0.392 | +50 | +40 | +26 | 5.63 | Frontal Pole |
|  |  |  | +44 | +44 | +20 | 4.79 | Frontal Pole |
|  |  |  | +38 | +40 | +32 | 4.46 | Frontal Pole |
|  |  |  | +40 | +46 | +32 | 4.22 | Frontal Pole |
| 2 | 55 vox | 0.688 | +40 | +22 | +4 | 5.32 | Frontal Operculum Cortex |

**Supplementary Table 6**. *TD specificity in cognitive effort* contrast ((TD task difficult > TD task easy) > (OD task difficult > OD task easy); p=0.001, k=50)).

| **Cluster** | **Cluster Size** | **Cluster-level p value (FWE-corrected)** | **Local Maxima** | | | | |
| --- | --- | --- | --- | --- | --- | --- | --- |
|  |  |  | **MNI coordinates** | | | **T-value** | **Anatomic Region** |
|  |  |  | X | Y | Z |  |  |
| 1 | 178 vox | 0.038 | -22 | -50 | -40 | 7.3 | Dorsal Dentate Nucleus of the Cerebellum |
|  |  |  | -32 | -50 | -40 | 5.54 | Dorsal Dentate Nucleus of the Cerebellum |
